# Application of Earth Pigments in Cycloolefin Copolymer: Protection against Combustion and Accelerated Aging in the Full Sunlight Spectrum

**DOI:** 10.3390/ma13153381

**Published:** 2020-07-30

**Authors:** Bolesław Szadkowski, Małgorzata Kuśmierek, Przemysław Rybiński, Witold Żukowski, Anna Marzec

**Affiliations:** 1 Institute of Polymer and Dye Technology, Faculty of Chemistry, Lodz University of Technology, Stefanowskiego 12/16, 90-924 Lodz, Poland; boleslaw.szadkowski@dokt.p.lodz.pl (B.S.); malgorzata.kusmierek@dokt.p.lodz.pl (M.K.); 2 Institute of Chemistry, the Jan Kochanowski University, Żeromskiego 5, 25-369 Kielce, Poland; przemyslaw.rybinski@ujk.edu.pl; 3 Department of Chemical Engineering and Technology, Cracow University of Technology, 31-155 Kraków, Poland; witold.zukowski@pk.edu.pl

**Keywords:** earth pigments, ochres, ethylene–norbornene copolymer, light stability, accelerated aging, flammability

## Abstract

In this paper, we assess various natural earth pigments as potential colorants and stabilizers for ethylene–norbornene copolymer composites. Several cycloolefin copolymer (COC) composites colored with 2 wt% of a selected pigment were prepared using a two-step mixing method. The aging resistance of the polymer composites was investigated in terms of changes to their mechanical properties, following accelerated aging in the full sunlight spectrum (100, 200, 300, 400, and 500 h). Fourier-transform infrared spectroscopy (FTIR), surface energy measurements, and spectrophotometry were used to assess the color changes, surface defects, and morphology of the composites. Thermogravimetric analysis (TGA) was used to study their thermal stability. The combustion characteristics of the prepared COC composites were evaluated based on the microcombustion calorimetry test (MCC). The application of earth pigments resulted in interesting color changes and a significant improvement in the aging resistance of the COC-filled samples, as evidenced by higher aging factor values and lower carbonyl index parameters compared to the reference (COC). The best results were observed for hematite (HM), gold ochre (GO), and red ochre (RO). In addition, the application of earth pigments, especially iron ochre (IO) and red ochre (RO), in COC contributed to a significant reduction in the heat release rate (HRR) values, indicating improved flame retardancy. This research opens the possibility of producing colorful COC composites with enhanced photostability and reduced flammability for use in polymer applications.

## 1. Introduction

Dyes and pigments are widely employed in various industrial and commercial applications, such as paints, construction materials, paper, ceramics, textiles, and plastics [1,2]. Pigments are colorants that have been incorporated into a polymer composite, via a dispersion process which forms a separate phase in the dyed material. Pigments have a limited tendency to migrate and are practically insoluble in most media, which is important from a practical point of view. Pigments are conventionally classified as either inorganic or organic. Organic pigments are in general characterized by high brightness and good color strength. However, they offer different fastness properties (low thermo- and photostability) [3]. Organic pigments require a lengthy synthesis process, often in the presence of harmful solvents, which is a disadvantage from both the ecological and economical points of view. There is therefore great interest in finding potential substitutes for organic pigments currently used in many areas of industry.

Recent studies have presented hybrid pigments with improved applicative properties, prepared by modifying inorganic carriers with organic dyes [4,5,6,7]. Another alternative is the application of inorganic pigments as colorants. The advantage of inorganic pigments is that they are produced via relatively simple chemical reactions (particularly oxidation) or found naturally as earths. Earth pigments are inorganic, naturally-occurring materials of mineral origin. They have been used as coloring substances for thousands of years, due to their widespread availability, strong coverage, durability, and color stability [8].

Iron oxides are a large group of pigments, including earth pigments, ochres, and iron ores. They are distinguished based on their color, for instance as yellow ochres, red ochres, and umbers. This may be a non-clay pigment, such as an iron oxide, or a chromogenous element in the clay structure [9]. Recently, earth pigments have attracted much interest, due to their intense color, low cost, facile application, and versatile properties [10,11,12,13,14]. They have found many applications, including as catalysts and drug carriers, in magnets and plastics, as well as for wastewater treatment and adsorption processes.

Many plastic products are used outdoors, so it is of great importance to study their chemical and physical behavior in outdoor environments. Photodegradation is known to cause serious alterations in the physical performance of materials, such as surface yellowing, cracking, embrittlement, and stiffening of the polymer composite [15,16]. Based on the literature on pigments in polymer systems, pigment colorants that absorb UV light could provide a shielding effect against photodegradation [17,18,19]. Iron ores (including earth pigments) can be used as additives in composite materials, to improve their functional properties. Camlibel et al. [14] showed that the incorporation of iron ores as additives in cotton fabrics coated with polyacrylate polymer improved their flame retardant and antibacterial properties, while also providing UV protection and coloration. Haug et al. [20] and Kiat-amnuay et al. [21] reported that the application of dry earth pigments can protect silicone rubber composites against weathering. Other research shows that hematite pigment improves thermal stability and reinforces the mechanical performance of polymer composites [22,23].

When selecting colorants for polymer blends, it is necessary to know the effect of the additive on both the polymer stability and its other applicative properties. The physico-chemical performance of earth pigments, such as high thermal stability and chemical resistance, as well as their natural origin, make them interesting colorants in polymer materials. There is little in the literature regarding the stabilization of polymer composites against long-term light exposure using earth pigments such as golden, iron or red ochre. Moreover, the wide variety of earth pigments and their complex chemical constitution may affect the polymer degradation process in different ways. The potential use of earth pigments as stabilizers for cycloolefin copolymers (COC) has never been discussed. These copolymers were first produced in the late 1950s but only became available on the market relatively recently. One of the most important is ethylene–norbornene copolymer, which is known for its high purity, glass-like transparency, and low permeability to gas and water. Due to these properties, ethylene–norbornene copolymer is widely used in packaging [24,25]. However, products made from COC exhibit significant photodegradation and thermal oxidation when exposed to outdoor conditions, resulting in alterations to their surface characteristics and deterioration of their mechanical properties. This currently precludes the long-term use of materials made from COC in outdoor environments.

In our previous work, we studied the stabilizing effects of organic-inorganic pigments based on magnesium-aluminum hydroxycarbonates and organic dyes on COC polymers under UV irradiation and aging in the full sun spectrum [26,27]. In the present study, we investigated the application of earth pigments as natural multicolor stabilizers for ethylene–norbornene copolymer. Earth pigments of different colors and compositions were applied as additives in the COC copolymer at 2% concentration. The resulting colorful composites were subjected to prolonged irradiation in the full sunlight spectrum. Alterations in their surface and color characteristics were assessed, as well as mechanical performance. Their effect on the flammability of the COC composites was also evaluated.

## 2. Materials and Methods

### 2.1. Materials

Six earth pigments of different colors were selected for this study (the pigments were in powder form and the size of the pigments was in range of 1–50 µm). The names, abbreviations, suppliers, and chemical compositions of the samples are listed in Table 1. Ethylene–norbornene random copolymer (ELASTOMER E-140) with 40 wt% bound norbornene content was purchased from IMCD Polska Sp. z o. o. (Warszawa, Poland) and employed as a polymer matrix. Diiodomethane and ethylene glycol were supplied by Sigma Aldrich (St. Louis, MO, USA). All chemicals were used as received, without further purification.

### 2.2. Experimental Methods

The compounding process was carried out on an internal Brabender Measuring Mixer N50 (Duisburg, Germany). The COC compounds contained 100 phr (parts per hundred part of rubber) of ethylene–norbornene copolymer and 2 phr of earth pigment. The ethylene–norbornene compositions were processed at a rotor speed of 50 rpm at an initial temperature of 110 °C for a mixing time of 10 min. Subsequently, the as-obtained COC compounds were mixed in a laboratory-scale open two-roll mixing mill. Polymer films with a thickness of 1.0 ± 0.1 mm were obtained by pressing the COC-filled compounds between two hot steel plates for 5 min at 110 °C and 6 MPa.

The resistance of the COC composites to photodegradation was determined on a Solar Climatic 340 instrument. Accelerated ageing was performed in a wavelength (λ) range of 280–3000 nm over 500 h, with light radiation intensity of 1200 W/m^2^ (luminous flux 110,000 lm). The polymer samples were mounted in specimen holders, so that their entire surface was exposed to light radiation. The weathering procedure consisted of two alternately repeating segments: day (solar irradiation, 70 °C, humidity 50%, 8 h) and night (20 °C, humidity 60%, 4 h). The color, surface, and mechanical properties of the prepared composites were characterized following the weathering process.

A Minolta CM-2500d spectrophotometer (Konika Minolta Sensing Inc., Osaka, Japan) was used to measure the color of the treated and untreated COC composites, in a spectral range from 360 to 740 nm. The CIE L∙a∙b color space system was employed to determine the surface color of the samples. The total color change parameter (∆E) was calculated as outlined in ISO 7724, according to the following equation:

(1) $$\Delta E=\sqrt{∆L^{2}+∆a^{2}+∆b^{2}},$$

where ∆L, ∆a, and ∆b represent the differences between the initial and final values of L (brightness), a (red-green color coordinate), and b (yellow-blue color coordinate), respectively.

The reflectance of the colored COC composites was recorded on an Evolution 201/220 UV-Visible Spectrophotometer (Thermo Fisher Scientific, Waltham, MA, USA). The measurements were performed for composite specimens that were placed in a special cell holder with a spectral window from 1100 to 200 nm at room temperature. Before the experiments, the baseline was corrected using a special calibration adapter. The accuracy of the apparatus was ±0.8 nm and the repeatability was ≤0.05 nm.

The mechanical performance of the samples before and after the aging process were evaluated based on tensile tests, using a Zwick 1435 tensile testing machine (Zwick Roell Group, Ulm, Germany) in accordance with the ISO-37 standard. The mechanical parameters were evaluated in the presence of the extensometer and the tensile strength value was determined from the equation:

T_S_ = F/W ∙t, (2)

where F is the force registered at break (N), W is the width of the narrowed part of the sample (mm), and t is the sample thickness (mm).

The measurements were carried out on dumbbell-shaped specimens at room temperature, with a crosshead speed of 500 mm/min. Five individual samples of each tested composite were used. Based on the results, the aging coefficient was calculated using the following formula [28]:

Aging factor = (T_S_∙E_B_)_after aging_/(T_S_ ∙E_B_)_before aging_, (3)

where T_S_ and E_B_ are the tensile strength (MPa) and elongation at break (%) of the COC composites.

The surface characteristics of the studied COC samples were examined using an OEC 15EC goniometer (DataPhysics Instruments GmbH, Germany). Surface free energy was determined based on the Owens, Wendt, Rabel, and Kaelble (OWRK) method [29]. The polar and disperse parts of the surface energy are described by the equations:

σ_l_ = σ_l_^d^ + σ_l_^p^, (4)

and

σ_s_ = σ_s_^d^ + σ_s_^p^, (5)

where σ_l_^d^ and σ_l_^p^ represent the disperse and polar parts of the liquid, while σ_s_^d^ and σ_s_^p^ represent the disperse and polar parts of the solid. The interfacial surface energy of the materials can be calculated according to OWRK from the contributions of the liquid and the solid, by finding the geometric mean and using the equations:

y = ax + b,(6)

and

(7) $$y=\frac{(1+cos\frac{\theta }{2})\cdot \sigma_{l}}{\sqrt{\sigma_{l}^{d}}};x=\sqrt{\frac{\sigma_{l}^{p}}{\sigma_{l}^{d}}};a=\sqrt{\sigma_{s}^{p}};b=\sqrt{\sigma_{s}^{d}},$$

To determine the contact angle value (θ), the following liquids with different polarities were employed: diiodomethane, ethylene glycol, and distilled water.

Fourier transform infrared (FTIR) spectroscopy was performed on the COC samples before and after weathering using a Thermo Scientific Nicolet 6700 FTIR spectrometer (Thermo Scientific, Waltham, MA, USA). The FTIR spectra for all the samples were recorded with 32 scans at 4 cm^−1^ resolution in a scanning range from 400 to 4000 cm^−1^. The carbonyl index (CI) was determined using the following equation [30]:

CI = A_C=O_/A _-CH2-_, (8)

where A_C=O_ refers to the area of the carbonyl absorption band in the range of 1800–1680 cm^−1^ and A_(-CH2-)_ is the area of the internal reference band in the range of 3000–2800 cm^−1^ corresponding to asymmetric stretching vibrations in methylene groups (this peak remains unchanged during photodegradation).

Thermogravimetric Analysis (TGA) in two different atmospheres was applied to monitor the thermal degradation process, which is related to mass loss as a function of rising temperature. A TA Instruments Q500 Thermogravimetric Analyzer (Mettler Toledo, Greifensee, Switzerland) was used with a heating rate of 10 °C/min, under argon (flow rate of 60 mL/min) and air (flow rate of 20 mL/min) atmospheres. Thermal stability tests were performed for samples with 10 mg mass in a temperature range of 25–600 °C.

Microscale combustion calorimetry was employed to determine the flammability of the composites filled with earth pigments. For this purpose, similar fragments of each composites weighing approximately 2.5 mg were analyzed on an MCC micro-calorimeter (Fire Testing Technology Limited). Flammability measurements were performed with the following parameters: pyrolyzer temperature 750 °C, combustor temperature 900 °C.

The morphology of the earth pigments and COC composites was assessed based on scanning electron microscopy (SEM). Microphotographs of the samples were obtained using a LEO1530 Gemini scanning electron microscope (Zeiss/Leo, Oberkochen, Germany). Prior to the SEM measurements, the composite samples were immersed in liquid nitrogen and the location of the fracture was covered with a carbon layer.

## 3. Results

### 3.1. Photostability

The degradation of polymer composites due to solar irradiation is a serious issue, resulting in numerous material failures, such as reductions in mechanical performance and deterioration of surface quality (yellowing and cracks). The resistance of a polymer to solar light is one of the main factors that determine the lifetime of the final polymeric product. The application of pigments with the ability to absorb and/or screen out UV light can therefore have a marked protective effect. At the same time, some dyes and pigments have a negative effect on the degradation and oxidation of polymers, accelerating the degradation process. Therefore, it is important to study how the origin, structure, and chemical composition of pigments can influence the photostability of polymers.

**Figure 1:**
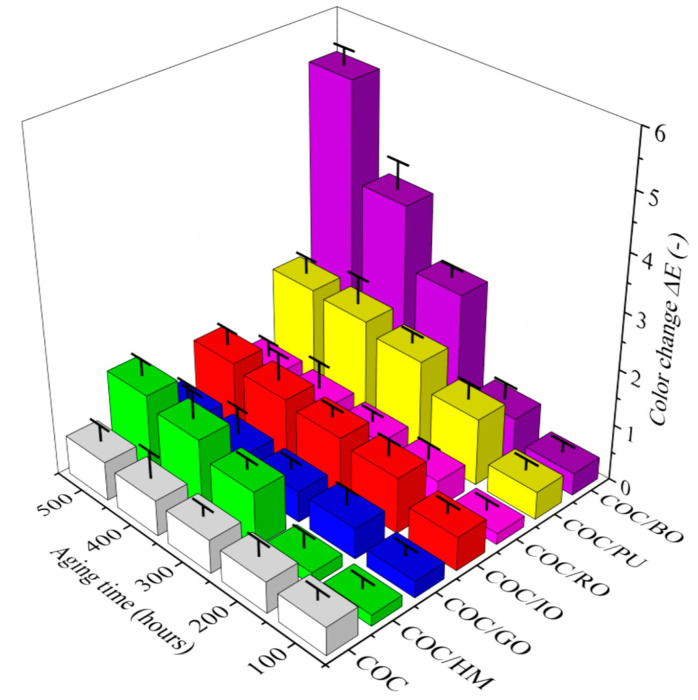
Color change parameter (ΔE) for the studied composites at different aging times.

The COC composites colored with various earth pigments were exposed to accelerated and prolonged solar aging. Their color, surface characteristics, and mechanical performance were monitored. Surface discoloration was represented by the total color change parameter (ΔE) registered after exposure of the samples to solar irradiation (Figure 1 and Table A1).

As would be expected, due to its transparency, the neat COC copolymer showed a negligible color change even after 500 h of aging. However, in most cases, there was only a slight increase in ΔE following exposure of the COC composites containing earth pigments to solar irradiation. Some slight discoloration was observed for COC/HM, COC/IO, and COC/PU after 300 h, while the surface color of the COC/GO and COC/RO composites remained unchanged even after 500 h of aging. Therefore, it can be concluded that the best protection against sunlight was provided by gold and red ochres.

The opposite effect was observed for brown ochre. The COC/BO composite showed a rapid change in ΔE after 300 h of aging. This difference indicates that the COC/BO composite faded much more than the other samples, probably due to the lower stability of the brown ochre pigment under the applied aging conditions. The solar heat energy generated in the NIR region (700–1100 nm) created heat waves, which contribute to the build-up of heat in the polymer and may result in faster degradation of this materials. Moreover, ultraviolet rays (UVA 400–320 nm and UVB 320–290 nm) cause a photochemical effect within the polymer structure which affects its stability. According to the literature, both organic and inorganic pigments generally improve the light stability of polymeric materials [31,32]. The positive impact of pigments on the light stability of polymer is most likely due to their screening or selective absorption of harmful radiation, and to the deactivation of polymer photoexcited species. However, several colorants are photoactive and can therefore catalyze and accelerate the photochemical breakdown of the polymer. Based on current knowledge, it is difficult to predict the extent to which a pigment will have a protective effect.

**Figure 2:**
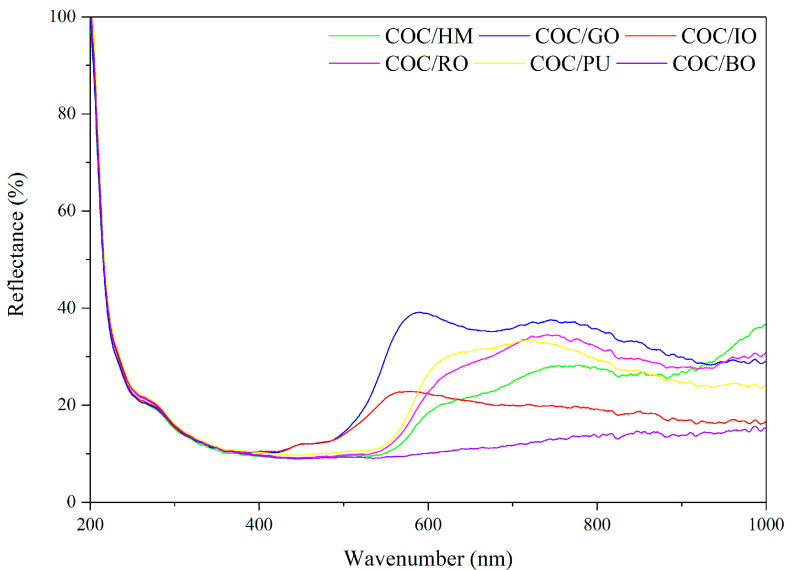
Light reflectance of the COC composites filled with different earth pigments.

The optical properties of the earth pigments were investigated using UV/Vis spectroscopy in the range of 200–1200 nm. Colored pigments have very different absorbance spectra, depending on the specific interaction of visible light with the valence electrons of the colorants. The yellow pigments (golen ochre and iron ochre) exhibited absorbance in range 200–400 nm, red COC composites (hematite, puzzola, and red ochre) were characterized by absorbance in higher values of wavelengths 300–600 nm, and sample with brown pigment showed wide, blurry peak in the UV and visible regions. Interestingly, NIR-reflectance values varied for particular pigments (Figure 2). The spectral results revealed the presence of brown ochre as colorant provide reflectance in NIR region lower than 10%. Therefore, it can be suspected that poor stability of COC/BO sample may be resulted from chemical nature of this pigment i.e., ability to absorption/reflection of light and its photoactivity in the studied irradiation condition [33].

The relatively small color changes in the COC composites filled with other earth pigments may be explained by the protective activity of the pigments in the polymer matrix, which physically block solar irradiation [34,35]. It is well-documented that iron oxides, which are also considered to be earth pigments, exhibit the ability to absorb light in a wide spectrum (200–800 nm) [36]. This may be the main reason earth pigments effectively protected the polymer composite surface against the negative effects of solar irradiation. In other words, under accelerated aging conditions, solar light may enter and damage unprotected COC materials; however, the application of earth pigments can prevent solar irradiation from photodegrading COC macromolecules, due to their absorption/reflection of damaging light radiation in the UV and NIR regions.

**Figure 3:**
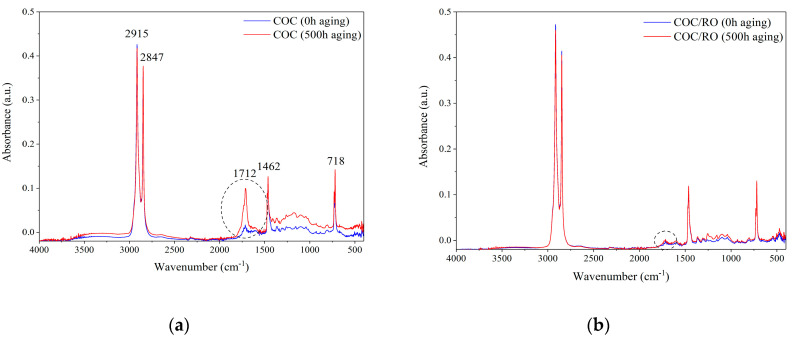
FTIR spectra of the COC copolymer composite (**a**) and COC/RO composite (**b**) before and after aging.

**Figure 4:**
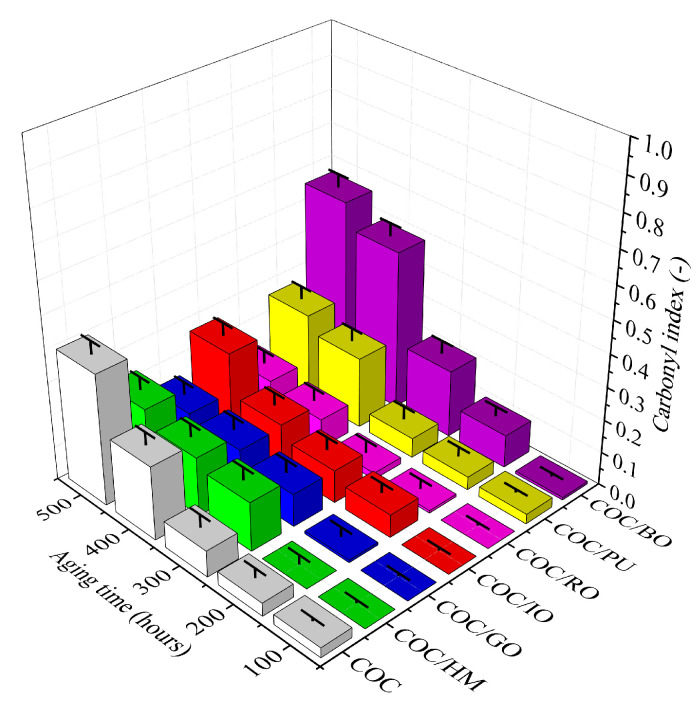
Carbonyl index (CI) parameters of the studied composites at different aging times.

The photodegradation progress was further examined using FTIR spectroscopy. Structural changes caused by exposure to solar irradiation were observed by monitoring the absorbance of the carbonyl groups (1600–1800 cm^−1^) on the FTIR spectra. The FTIR spectra of the COC composites show several characteristic peaks, such as 2915 and 2847 cm^−1^, which can be attributed to the stretching vibration of CH_2_; 1462 cm^−1^, which is related to the stretching vibration of C-O-C; and 718 cm^−1^, which can be assigned to CH_2_ rocking vibration (Figure 3). The most interesting result was observed at 1712 cm^−1^, which is attributed to the carbonyl groups (C=O). Neat COC exhibited a significant increase in this peak after aging, compared to the COC/RO sample. The CI parameters for all the studied composites were calculated based on Equation (8). The results are presented in Figure 4 and Table A2.

In the initial state of photodegradation (after 100 h), the CI values were different from zero and similar for all the compounds. However, the CI values started to increase gradually with exposure to solar light, indicating progressive degradation of the macromolecules. As shown in Figure 4, the CI of the COC copolymer increased from 0.05 (100 h) to 0.4 after 500 h of irradiation, indicating that numerous carbonyl groups were generated on the surface of the unfilled COC. This can be explained by radical processes accelerated by UV light, and it was reflected by the low stability of the unprotected COC. The most pronounced increase in the CI parameter was observed for neat COC and COC/BO, as their values after 500 h of aging were even double those of the other compounds. This means that neat COC is not resistant against prolonged exposure to solar light, whereas the brown ochre pigment may act as a pro-oxidant that accelerates the photodegradation of COC. In comparison, the other composites containing earth pigments were characterized by significantly slower and above all smaller increases in CI, which proves that these pigments had a positive effect on the stability of COC exposed to solar light radiation. Most of the earth pigments protected the COC more effectively against solar irradiation than the lawson-based pigment studied in our previous work [27]. The aging coefficients of the COC/Golden Ochre pigment showed values of around 1 after 300 h of irradiation, whereas the COC copolymer containing organic–inorganic pigment was found to be around 0.8 after a similar duration of aging treatment.

**Figure 5:**
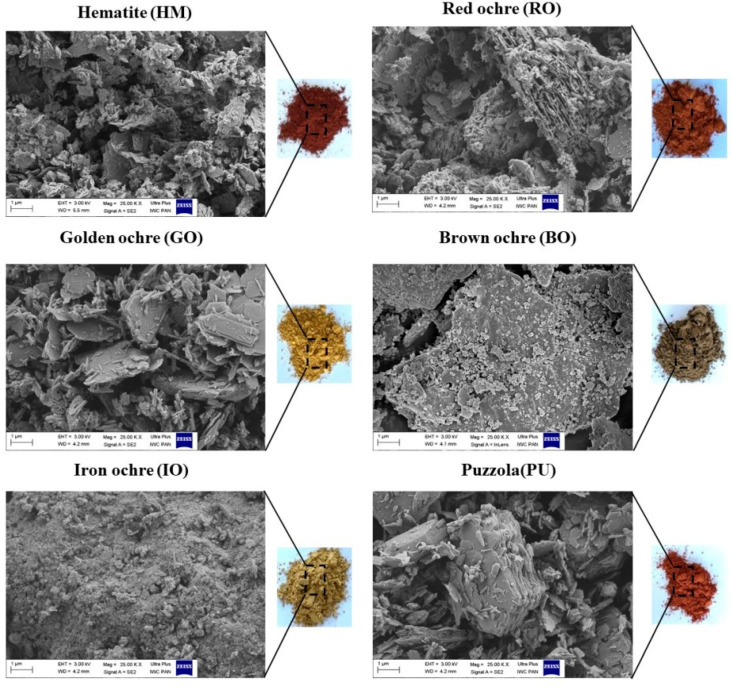
Scanning electron microscopy (SEM) images and digital photographs of the earth pigment powders.

**Figure 6:**
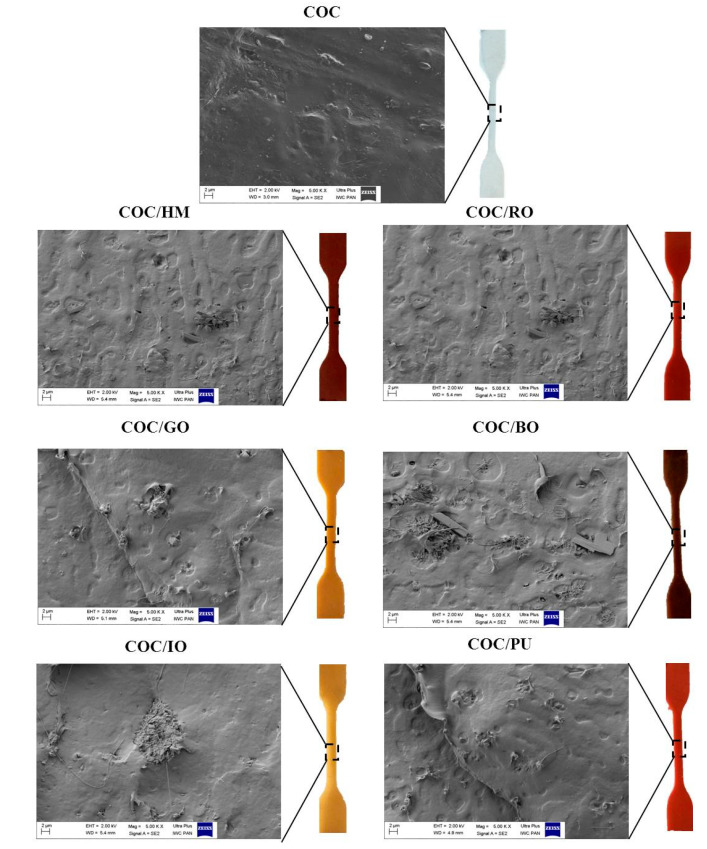
Scanning electron microscopy (SEM) images and digital photographs of the COC copolymer containing earth pigments.

A homogenous distribution of pigment is important for ensuring uniform color and good mechanical properties. It is known that aggregates/agglomerates can act as stress concentrators in polymer materials, which may therefore be weaker than composites containing well-dispersed particles. Scanning electron microscopy (SEM) was used to determine the morphology of the applied earth pigments and their distribution in the COC matrix. Figure 5 shows digital photographs and SEM images of the earth pigment powders. Figure 6 shows SEM images and digital photographs of the COC copolymer with different earth pigments.

Owing to the irregular nature of the fracture surface, it is difficult to describe the precise morphology of the pigments. However, the hematite (HM), gold ochre (GO), and brown ochre (BO) pigments clearly consisted of different types of particles (irregular plate-like and brick-like shapes), ranging from 0.5 up to a few μm, which often formed aggregates. The iron ochre (IO) particles were shaped similarly to the gold and brown ochre particles. The SEM photographs show the IO powder to be composed of a mixture of individual platelets and some stacks of platelets, with a wide particle size distribution from 1 to >5 µm. The iron ochre IO and puzzola (PU) pigments consisted of irregular, roughly shaped grains of varying dimensions, from 0.5 up to several microns.

The SEM images of cross-sections of the COC/pigment composites (Figure 6) show a homogenous distribution of colorant particles of rather uniform size. Morphology analysis revealed that some of the pigment particles were randomly dispersed in the COC copolymer, forming island-like agglomerates ranging in size from 2 to ~5 µm. Despite the presence of some regions with higher concentrations of colorant, digital photographs of the polymer materials colored with 2 wt% of the selected pigments show uniform color. Moreover, the results of mechanical tests were similar for the COC/pigment composites and the reference sample (COC). These observations imply that the amount of colorant applied in this study is sufficient to achieve the desired color, while simultaneously preserving the initial strength of the polymer.

The progressive degradation of macromolecules under outdoor conditions is most visible in the sudden deterioration of the physical properties of composites. Therefore, the mechanical properties of the COC-filled compounds were evaluated before and after the aging process. The results are presented in Table 2.

**Figure 7:**
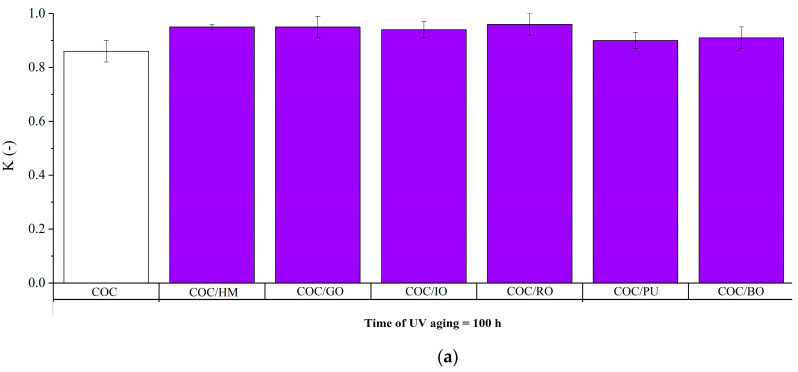

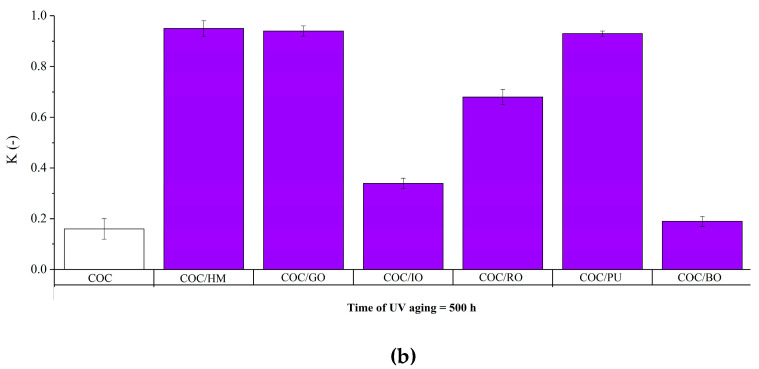
Aging factor (K) of the studied composites at different aging times: (**a**) 100 h; and (**b**) 500 h.

The pure COC composites reached tensile strength values of about 40 MPa. Similar results have been obtained previously by other authors for the same COC copolymer [30,37,38]. Exposure to solar irradiation was found reduce the tensile strength and elongation at break of the composites. In the cases of unprotected COC and COC/BO composites, these reductions were significant. This confirmed our previous observations. Along with the reduction of elongation at break, the increase in the modulus also testified to their advanced degradation. Previous reports in the literature have similarly noted a worsening of the mechanical properties of ethylene–norbornene copolymer after exposure to UV radiation [39,40], suggesting that COC may also not be resistant to prolonged exposure to UV radiation. However, our results indicate that the application of earth pigments may protect COC against the negative effects of solar irradiation, since the tensile strength and elongation at break parameters of the other COC-filled compounds remained almost unchanged. As an indication of the effect of the earth pigments the on the mechanical properties of the aged COC composites, the aging coefficient (K) determined for each sample is presented in Figure 7.

When the K value was close to 1, the composite was resistant to unfavorable conditions and its mechanical properties remained stable. However, when the K value was closer to 0, the composites underwent significant degradation. Based on our results, it can be concluded that after 100 h of aging the mechanical properties of all the composites were unchanged, but after 500 h the K values for COC, COC/IO, and COC/BO reduced significantly. Nonetheless, the K parameters for most of the composites with different pigments remained almost unchanged after 500 h of aging. Based on the mechanical changes following exposure to solar light, the protection efficiencies of the individual earth pigments can be summarized as follows: HM > GO > PU > RO > IO > BO. These results are in good agreement with those obtained for surface discoloration and the CI.

**Figure 8:**
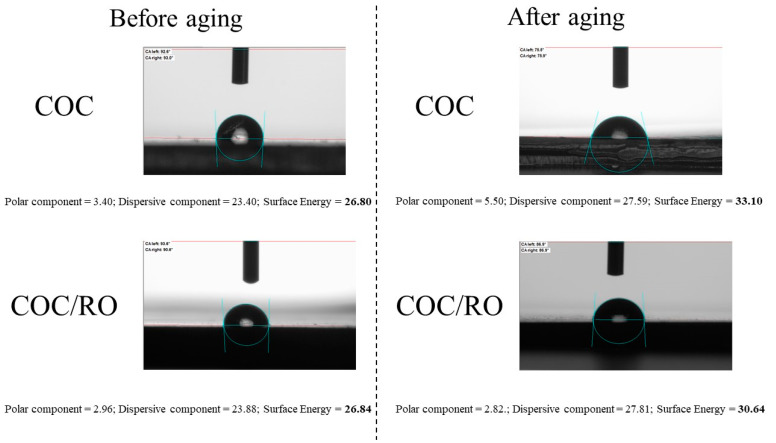
Surface energy of neat COC composite and COC filled with red ochre before and after 500 h of irradiation (images show the contact angle for water).

In the next part of study, the total surface energy and its polar and dispersive components were calculated from the contact angles (Figure 8).

In the case of pure COC, the dispersive (23.40 mJ/m^2^) and polar (3.40 mJ/m^2^) components of surface energy after 500 h of aging increased rapidly, up to 27.59 and 5.50 mJ/m^2^, respectively. The total surface energy of the COC sample changed in a similar way, increasing from 26.80 to 33.10 mJ/m^2^ as result of irradiation. After the addition of red ochre pigment, the polar and dispersive components of the COC/RO surface energy changed slightly in comparison to the reference sample. After 500 h of aging, the polar component remained almost unchanged, whereas the dispersive components rose from 23.88 to 27.81 mJ/m^2^. From these results, it can be concluded that during aging more polar groups were generated on the surface of the reference sample, resulting in a larger increase in surface energy compared to the sample with red ochre pigment.

### 3.2. Thermal Stability and Flammability

**Figure 9:**
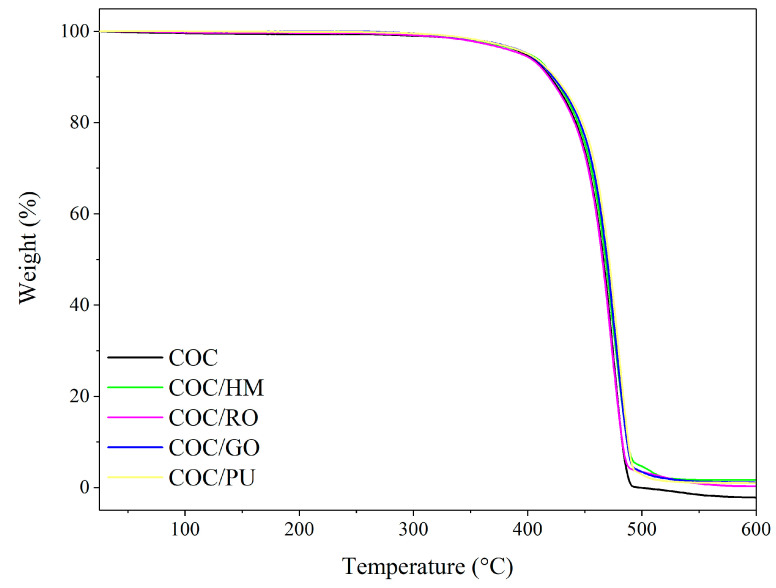
Thermogravimetric (TGA) curves obtained for COC composites filled with different earth pigments measured in an inert atmosphere.

**Figure 10:**
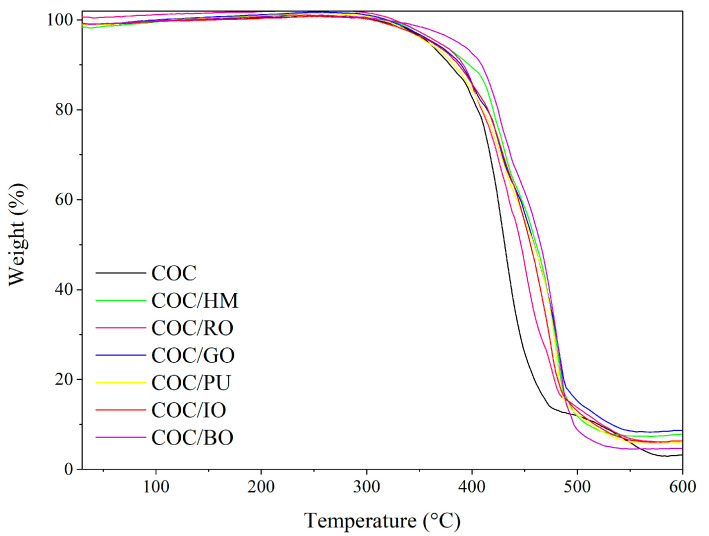
TGA curves obtained for COC composites filled with different earth pigments measured in air atmosphere.

**Figure A1:**
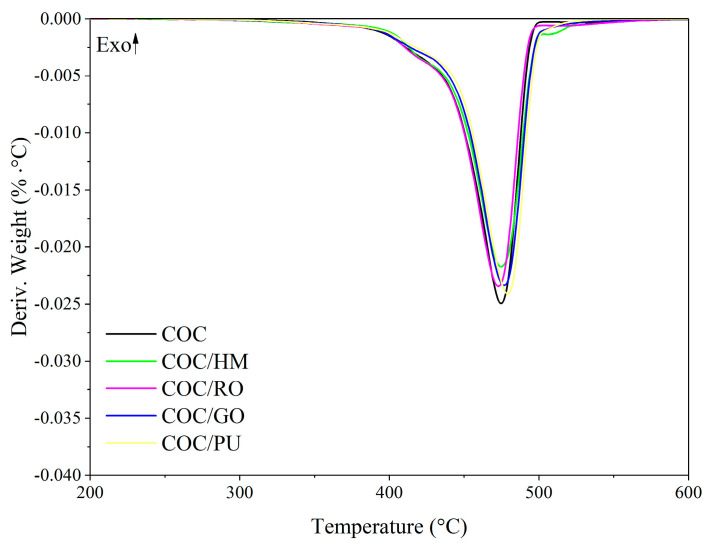
Derivate thermogravimetric (DTG) curves obtained for COC composites filled with different earth pigments measured in an inert atmosphere.

**Figure A2:**
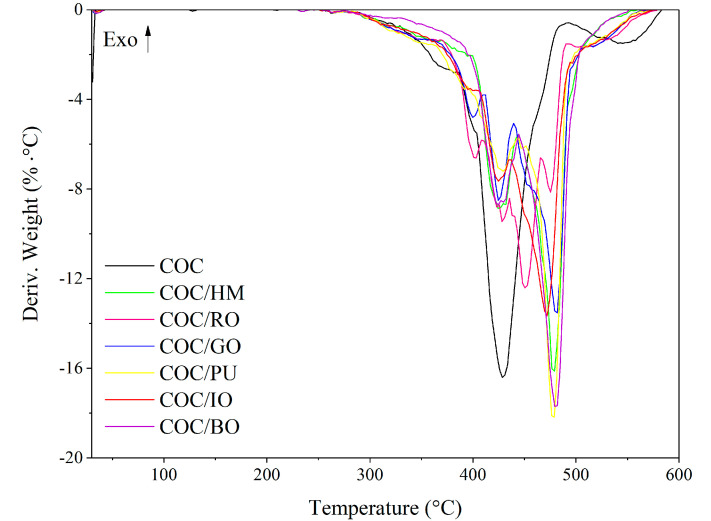
Derivate thermogravimetric (DTG) curves obtained for COC composites filled with different earth pigments measured in an air atmosphere.

Figure 9, Figure 10, Figure A1 and Figure A2 show thermogravimetric (TGA) and derivate thermogravimetric (DTG) curves for pure COC and COC loaded with 2 phr of different earth pigments, measured in different atmospheres. Table 3 summarizes thermal degradation temperatures corresponding to 5% (T_05%_), 20% (T_20%_), and 50% weight loss (T_50%_). From the TGA data in Figure 9, it can be observed that there was hardly any difference between the considered composites in terms of thermal stability measured in an inert atmosphere. However, the T_20%_ and T_50%_ of pure COC were slightly lower than those for COC containing puzzola pigment. This was most likely due to the presence of different metals and salts in the chemical composition of this pigment, which may enhance the resistance of COC composites to thermal decomposition. The TGA measurements presented in Figure 10 show that incorporation of earth pigments enhanced the stability of the COC composites at elevated temperatures in the presence of air. The T_50%_ parameter of the COC-filled composites in an air atmosphere was up to 30 °C higher than that for pure COC.

**Figure 11:**
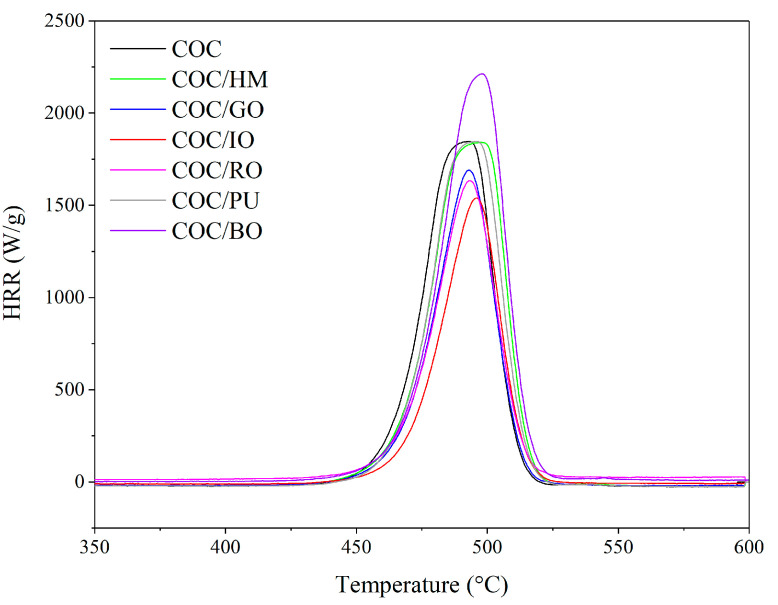
Heat release rate versus temperature obtained for COC composites with different earth pigments.

The effect of the different earth pigments on the combustion of the COC copolymer was investigated by microscale combustion calorimetry (MCC). This test is now commonly used as a bench-scale method for investigating the combustion behavior of different polymeric materials [41,42]. The MCC test provides important parameters for determining the flame-retardant properties of the materials, such as the heat release rate (HRR), total heat release (THR), and heat release capacity (HRC). The heat release rate (HRR) curves of the COC composites obtained from MCC tests are presented in Figure 11. The corresponding data are listed in Table 4.

It is important to note that the earth pigments reduced the fire hazard of the COC, in terms of the HRR, which is a propelling force of fire [43]. As can be seen in Figure 11, the HRR peak for the neat copolymer was higher than most of the COC composites filled with earth pigments. This means that the application of earth pigments may reduce the flammability of COC composite materials. On the other hand, the use of BO had the opposite effect, indicating that brown ochre may support combustion of COC to some extent. Regardless of the chemical structure of the earth pigments, in most cases, the application of pigments as colorants for COC clearly reduced both the total heat release and the heat release capacity of the composites. It should be emphasized that the amount of the pigments used was only 2 phr. The most pronounced reduction in the flammability parameters was observed for COC composite filled with iron ochre. In this case, the THR and HRC parameters of the COC compound were reduced after the application of IO from 79 and 1851 (neat COC) to 43 and 1530 J/gK (COC/IO), respectively. In general, the flammability of the COC composites filled with various earth pigments can be ordered as follows, starting from the least flammable: COC/IO < COC/RO < COC/GO < COC/HM < COC/PU < COC < COC/BO. The improved flame safety of the COC composites upon the incorporation of earth pigments may result from the presence in their structure of metal oxides, including transition metals. Generally, most transition-group metals possess catalytic activity and can alter the rates of processes including thermal degradation noticeably. Iron oxides that enter the gas zone are treated as free radical scavengers, interrupting the high-energy reactions that occur in this zone. Previous studies [44,45,46] have shown that the incorporation of fillers containing metal ions, including mineral pigments, may have a considerable influence on the fire hazard characteristics of polymer composites. In the present work, we also observed that earth pigments containing transition metal oxides can reduce COC flammability.

## 4. Conclusions

In this study, we produced a series of new multicolor ethylene–norbornene composites by introducing various natural earth pigments into COC copolymer via melt mixing. The composites were subjected to artificial solar ageing. The characteristics of the composites after exposure to sunlight were examined by Fourier transform-infrared spectroscopy, surface energy measurement, mechanical tests, and scanning electron microscopy. The earth pigments were found to have a protective effect on the polymer matrix, by comparison to the pure copolymer. The COC-filled composites were less susceptible to degradation compared to the uncolored copolymer, as evidenced by negligible changes in their color characteristics and surface energy, as well as by their very high ageing factors after 100, 200, 300, 400, and 500 h of aging in the full sunlight spectrum. It can be concluded that the earth pigments effectively absorbed part of the radiation, stabilizing the whole composite. Moreover, thermogravimetric and microcombustion calorimetry analyses revealed that the COC composites colored with earth pigments remained stable when subjected to elevated temperatures and their resistant to flame was improved. These comprehensive studies suggest that the application of multicolor earth pigments such as hematite, gold ochre, and red ochre may be an effective way to protect cycloolefin copolymer against the negative effects of solar irradiation, while iron and red ochres may also strongly enhance the flame retardancy of colored COC. These results open the way for the preparation of multicolor cycloolefin composites with improved light stability and flame retardancy, which could be used in packaging materials.

## Figures and Tables

**Table 1:** Description of earth pigments applied in the study.

Name	Abbreviation	Supplier	Chemical Composition
Hematite *	HM	Kremer Pigments	Fe_2_O_3_
Gold ochre *	GO	Kremer Pigments	Fe_2_O_3_, SiO_2_, Al_2_O_3_, CaCO_3_
Iron ochre *	IO	Kremer Pigments	Fe_2_O_3_·H_2_O, Fe_2_O_3_, Al_2_O_3_, CaCO_3_, SiO_4_
Red ochre *	RO	Kremer Pigments	Fe_2_O_3_, SiO_2_, Al_2_O_3_
Brown ochre *	BO	Kremer Pigments	Fe_2_O_3_, Al_2_O_3_, Mn_2_O_3_, SiO_4_, CaCO_3_
Puzzola	PU	Kremer Pigments	Mix of red earths containing Sb, As, Ba, Be, Pb, Cd, Cr, Co, Cu, Mn, Ni, Os, Hg, Se, Au, Tl, V, Sn, Zn.

* Natural origin.

**Table 2:** Mechanical parameters of the studied composites in their initial state and after 500 h of aging (standard deviation of Ts ± 0.7 MPa, S_E100%_ ± 0.6 MPa, E_B_ ± 45%).

Composite Name		T_S_ (MPa)	S_E100%_ (MPa)	E_B_ (%)
COC	Before aging	40.5	9.5	802
	After aging	12.7	10.3	415
COC/HM	Before aging	41.5	9.4	819
	After aging	40.2	8.7	801
COC/GO	Before aging	40.4	9.8	809
	After aging	39.9	8.5	771
COC/IO	Before aging	41.4	9.3	831
	After aging	21.3	8.8	556
COC/RO	Before aging	42.4	9.7	859
	After aging	34.4	8.6	717
COC/BO	Before aging	41.7	9.4	807
	After aging	15.2	10.2	410
COC/PU	Before aging	41.7	9.7	826
	After aging	39.0	8.6	822

T_S_, tensile strength; S_E100%_, stress at 100% elongation; E_B_, elongation at break.

**Table 3:** Thermal decomposition temperatures for COC composites filled with different earth pigments (standard deviation of T_05,20,50%_ = ±3 °C).

Composite Name	T_05%_ (°C)	T_20%_ (°C)	T_50%_ (°C)
**Argon atmosphere**			
COC	398	442	466
COC/HM	401	444	468
COC/GO	400	446	470
COC/RO	398	442	465
COC/PU	400	449	471
**Air atmosphere**			
COC	359	406	431
COC/HM	370	422	462
COC/GO	365	415	460
COC/RO	368	411	448
COC/PU	359	412	459

T_05%_, T_20%_, T_50%_, thermal decomposition temperatures degrading 5%, 20%, and 50% of sample, respectively.

**Table 4:** Microscale combustion calorimetry analysis data.

Compound	HRR (W/g)	THR (kJ/g)	HRC (J/gK)
COC	1857 ± 93	79 ± 4	1851 ± 93
COC/HM	1839 ± 93	64 ± 3	1845 ± 92
COC/GO	1685 ± 84	49 ± 3	1620 ± 81
COC/IO	1526 ± 76	43 ± 2	1530 ± 77
COC/RO	1633 ± 82	47 ± 2	1599 ± 80
COC/BO	2210 ± 111	67 ± 3	2188 ± 109
COC/PU	1843 ± 92	61 ± 3	1853 ± 93

HRR, heat release rate; THR, total heat release; HRC, heat release capacity.

## Appendix A

**Table A1:** Total color change (∆E) parameter of samples exposed to different aging times.

Compound	∆E				
	100 h	200 h	300 h	400 h	500 h
COC	0.50	0.56	0.60	0.64	0.70
COC/HM	0.20	0.23	1.00	1.30	1.50
COC/GO	0.30	0.55	0.56	0.60	0.66
COC/IO	0.60	1.00	1.10	1.20	1.30
COC/RO	0.20	0.50	0.60	0.65	0.70
COC/BO	0.40	0.80	2.50	3.59	5.32
COC/PU	0.50	1.20	1.67	1.86	1.92

**Table A2:** Carbonyl index (CI) parameter of samples exposed to different aging times.

Compound	CI				
	100 h	200 h	300 h	400 h	500 h
COC	0.03	0.04	0.08	0.23	0.40
COC/HM	0.00	0.00	0.15	0.18	0.23
COC/GO	0.00	0.01	0.10	0.13	0.15
COC/IO	0.00	0.07	0.10	0.14	0.27
COC/RO	0.00	0.01	0.02	0.09	0.11
COC/BO	0.01	0.10	0.21	0.49	0.56
COC/PU	0.03	0.04	0.06	0.22	0.27
